# Staying connected in a changing world: home-dwelling older adults experiences of belonging, well-being, and barriers to social life

**DOI:** 10.3389/fpubh.2026.1923947

**Published:** 2026-09-25

**Authors:** Therese Hugøy, Grethe Eilertsen, Helle K. Falkenberg, Anette Hansen

**Affiliations:** 1 Faculty of Health and Social Sciences, University of South-Eastern Norway, Porsgrunn, Norway; 2 USN Research Group of Older Peoples’ Health, University of South-Eastern Norway, Drammen, Norway; 3 Faculty of Health and Social Sciences, University of South-Eastern Norway, Drammen, Norway; 4 National Centre for Optics, Vision and Eye Care, Faculty of Health and Social Sciences, University of South-Eastern Norway, Kongsberg, Norway

**Keywords:** aging, aging in place, home care nursing, home-dwelling, living at home, loneliness, qualitative study, social life

## Abstract

**Background:**

As “aging in place” becomes the global policy standard, understanding the social dimensions of living long lives at home is crucial. While the significance of remaining at home is well-documented, we lack insight into how health challenges and the receipt of home care nursing reshape older adults’ social experiences. This study explores the experiences of social life in the context of aging in place among older home-dwelling people receiving home care services, aiming to provide a nuanced perspective on what it means to be socially connected.

**Methods:**

A qualitative study was conducted in two Norwegian municipalities. Interviewing 24 older home-dwelling adults aged 75 + receiving home care nursing. Data were analyzed using reflexive thematic analysis.

**Results:**

Social life was experienced as a dynamic and heterogeneous phenomenon, deeply rooted in personality and local context. Three main themes emerged: *Side by side, worlds apart: the pursuit of belonging in changing times*. The local environment acted either as a vital haven or a source of alienation as social circles diminished. *Quality over quantity: Prioritizing meaningful connections over social quantity.* Participants prioritized meaningful, deep connections over frequent social interactions, emphasizing a sense of belonging and well-being. *From ease to effort: navigating barriers to social support*. Maintaining a social life required significant effort due to health-related limitations. Participants balanced the desire for autonomy with an increasing need for support to remain socially integrated.

**Conclusion:**

Well-being in changing times hinged on the local environment, experienced through the interplay between the neighborhood as a safe haven and an arena of disconnection. Within these environments, participants prioritized meaningful engagement over volume of social contacts; however, health-related barriers made it increasingly hard to maintain a social life. Supporting social engagement in later life necessitates more than merely encouraging individual initiative. A nuanced understanding of aging must account for the fact that individual capacity cannot be understood in isolation from the social and material conditions that sustain it. Clinicians and home care services should differentiate between desired solitude and involuntary loneliness to foster belonging, well-being, and a meaningful social life.

## Introduction

While “aging in place” is the prevailing global health paradigm, the shift toward home-based care creates new demands for understanding how older adults with health challenges sustain their social lives within these settings. This trend is driven by a convergence of factors: the personal desire of older adults to maintain autonomy in familiar surroundings and the institutional prioritization of home-based care. The concept is central to international health policies, which posit that continuity of place and the preservation of independence are essential for well-being in later years (1). From a governmental perspective, this emphasis is further reinforced by the cost-effectiveness of home care relative to institutional alternatives, an economic imperative that underscores the current global shift toward home-based service models (2).

While aging in place is associated with autonomy and familiarity, it can also present significant challenges for health and well-being. As people age, many experience declines in health and functional capacity, resulting in increased reliance on both formal and informal support (3). Aging in place can make individuals more vulnerable to social isolation and loneliness, phenomena consistently linked to poorer health outcomes such as depression, cognitive decline, and increased mortality (4, 5). Social connection is widely recognized as a cornerstone of health across the lifespan. Research over several decades has demonstrated that both the presence and the quality of social relationships contribute to lower morbidity and mortality rates and play a protective role against psychological distress (6–11).

Recent studies provide new knowledge on the subjective significance of social interaction in later life. For many older adults, personal connections offer understanding, validation, and a sense of continuity with the self (12, 13). The capacity to express inner thoughts and share everyday experiences is described as essential for emotional well-being and the prevention of loneliness. Well-being in old age is therefore not limited to the absence of illness but is bound up with sustaining meaningful connections, belonging, and everyday practices that affirm identity and purpose (14, 15).

One of the challenges associated with a reduced social life for home-dwelling adults is loneliness, defined as *“a subjective and distressing emotional state that occurs when there is a perceived mismatch between an individual’s actual social relations and their desired level of connection”* (16) p 2. This phenomenon is not only shaped by personal expectations but also intricately linked to various dimensions of sociality, including the number of contacts (structural), the support received (functional), and the overall depth of these interactions (17–19).

Diverse networks that include family, friends, and other significant ties appear especially beneficial, while restricted networks or unreciprocated ties may heighten vulnerability to loneliness (20). Moreover, the association between social connections and mental health is reciprocal; strong ties can buffer against depression; however, depressive symptoms themselves may lead to withdrawal and a gradual contraction of one’s social world (20).

Loneliness in later life is increasingly recognized not only as a social or emotional condition but also as a deeply existential experience, particularly among older adults receiving home care (13). For many older adults, the inherent need to belong remains a primary concern; however, this dimension of their lives is frequently jeopardized as advancing age often leads to a contraction of social networks and a subsequent reduction in meaningful social connections (21, 22).

Social engagement in later life is deeply contextual, shaped by the interplay between individual strategies and structural environments. Research on age-friendly neighborhoods and communities (12, 13) emphasizes that social cohesion, rooted in trust and mutual support, is a key determinant of well-being that can buffer against loneliness. Creating such environments requires integrating physical infrastructure, such as accessible housing and transport, with social qualities such as inclusion and respect. These dimensions are mutually reinforcing, where the material and social conditions of the local environment work together to sustain older adults’ belonging and participation (12, 13). Moreover, white papers (2) point to participatory and collaborative governance as essential, with older adults playing an active role in shaping the communities in which they live. These emerging ideals underscore a shift from viewing older adults as service recipients to recognizing them as co-producers of supportive community environments (2).

Research on aging in place in Norway reveals substantial variation in how policies are locally implemented (23). Organizational structures, staffing patterns, and environmental design can all influence the extent to which community-dwelling older adults are supported in maintaining social engagement (24). Although research has established strong associations between social isolation, loneliness, and adverse health outcomes, there remains relatively limited knowledge about how older adults receiving home care nursing experience and navigate their social lives.

This study is situated within the context of “aging in place”. While the WHO (1) defines the term as the ability to live in one’s home and community safely and independently, qualitative research emphasizes deeper dimensions. Wiles, Leibing (15) argue that aging in place is not merely “staying put” but a dynamic process involving identity, belonging, and social connectivity. By adopting this broader perspective, the study moves beyond the logistics of home-based care to explore how the interplay between the individual and their familiar environment shapes the lived experience of social life in later years.

By focusing on older adults’ own perspectives, this study aims to explore how home-dwelling older adults receiving home care experience and make sense of their social lives within the context of aging in place. Attention is given to how they interpreted opportunities and barriers for social connection in light of personal, relational, and contextual circumstances.

## Methods

A qualitative approach using open interviews was employed to develop new knowledge to deepen the understanding of how older home-dwelling adults receiving home care nursing experience their social lives and further exploring the significance of these experiences for their health and well-being.

### Setting

The present study focuses on exploring the perspective of older adults themselves and was conducted in two municipalities in Southeastern Norway: one rural and one semi-urban. In Norway, the national government holds responsibility for ensuring equitable access to healthcare services for all citizens, independent of economic status, social status, or geographical location (25). The organization and delivery of primary care are delegated to the municipal level. Whithin municipalities, home care services are divided into home care nursing and home care help. Home care nursing, such as assistance with medication management and wound care, is provided free of charge, whereas home care help, including household maintenance, is subject to a means-tested fee (25). Access to home care services is based on individual assessments of need and granted in accordance with municipal guidelines.

The present study is part of a larger research project entitled “More Good Days at Home” (RCN number 320622), which aims to advance health-promoting practices in municipal healthcare services for older recipients of home care services.

### Recruitment and participants

Participants were recruited according to the following inclusion criteria: age 75 years or older, receiving home care nursing for a minimum of 1 year, and the ability to take part in an interview of approximately 1-h duration.

A purposeful sampling strategy was applied to achieve variation across characteristics considered relevant to the aim of the study (26). Diversity was sought with regard to gender, marital status (married, single, or widowed), living arrangement (alone or cohabiting), having or not having children, and type of residence (detached house or apartment). Efforts were also made to include ethnic diversity; however, this was not feasible as few older adults with immigrant backgrounds received home care nursing in the participating municipalities.

Participants were recruited by nurses in the home care services, who provided eligible individuals with oral and written information about the study. Those who expressed interest consented to having their contact details forwarded to the first author, who subsequently arranged the interviews. Written informed consent was obtained from all participants prior to participation. The sample comprised 24 participants, ranging in age from 75 to 103 years, which include 15 women and 9 men (Table 1).

**Table 1:** Participant characteristics (*N* = 24).

Characteristic	Category	*N* (%)
Age (years)	75–80	6 (25%)
	80–90	9 (38%)
	90+	9 (38%)
Gender	Women	15 (63%)
	Men	9 (38%)
Marital status	Married/cohabiting	6 (25%)
	Widowed	14 (58%)
	Divorced/separated	2 (8%)
	Never married	2 (8%)
Living situation	Living alone	18 (75%)
	Cohabiting	6 (25%)
Housing type	House	15 (63%)
	Senior apartment	4 (17%)
	Apartment	5 (21%)
Informal caregivers	Children	19 (79%)
	Spouse	1 (4%)
	Both children and spouse	3 (13%)
	None*	4 (17%)
Home care visits per day**	Less than 1 per day	3 (13%)
	1–2	14 (58%)
	3–4	6 (25%)
	5 or more	1 (4%)
Residential area	Rural	12 (50%)
	Semi-urban	5 (21%)
	Urban	7 (29%)

Percentages may not total 100 due to rounding. *None: This includes both participants without next of kin and with relatives who do not provide care. **Self reported number of visits at the time of the interview. Several had previously received more or less frequent visits, as their health condition and needs fluctuated. Many had agreements with home care services and could call for assistance in case of acute needs.

### Qualitative interviews

Data used in this study were generated through qualitative interviews conducted as part of two previous qualitative studies in this project (3). The two studies included qualitative interviews with 10 and 14 participants, respectively, all of whom were older adults living at home and receiving home care nursing. The first study explored participants’ understandings of “home” from a health-promoting perspective (3), while the second focused on aspects contributing to well-being in everyday life, described as “good days” at home, as well as challenges related to maintaining quality of life in a home setting. Although social life was not an explicit focus of the original interview guides, the open-ended and qualitative approach encouraged participants to reflect broadly on their lived experiences, including social relationships and everyday social engagement. Therefore, the extent to which participants were asked follow-up questions about their social lives varied across interviews. This may have led to variations in the depth and richness of participant accounts. Consequently, the findings represent a nuanced interpretation of how social life and loneliness surfaced in the interviews, rather than a comprehensive assessment of these experiences. Nevertheless, the fact that these issues emerged spontaneously in interviews not specifically focused on loneliness may also indicate their relevance to participants’ everyday experience of aging in place.

The interview guides were developed based on thorough reviews of the literature. Each guide underwent discussion with the coauthors and nurses in the home care services and was subsequently pilot tested with an older adult. This process resulted in minor revisions to enhance the clarity of specific questions.

The first author conducted all 24 interviews. The interviews took place in the participants’ own homes. Conducting the interviews in participants’ homes provided contextual insights and situated their lives within everyday living conditions (27). The interviews were audio recorded, transcribed verbatim, and lasted between 40 and 120 min.

### Reflexive thematic analysis

A reflexive thematic analysis informed by Braun and Clarke was used (28). This approach explicitly foregrounds the researcher’s active and reflexive role in the analytic process, recognizing that themes do not simply emerge from the data but are generated through an interpretative engagement with the material. All interview transcripts were imported into NVivo software. To facilitate familiarization with the social aspects of the material (Phase 1), the data were re-read multiple times with a specific focus on the participants’ experiences and reflections regarding their social lives. Subsequently, the coding process (Phase 2) was initiated, during which segments of the text were systematically labeled considering the participants’ narratives about social engagement, the absence of social life, and their personal reflections on these phenomena. The coding was both interpretive and descriptive, with a main focus on interpretation (29). Once the coding was completed, the codes were organized into broader patterns of meaning to identify potential themes (Phase 3). This involved a recursive process of clustering-related codes that captured significant aspects of the participants’ social experiences and their implications for everyday life, health, and well-being. The analytically derived themes were then reviewed against the entire dataset to ensure they accurately represented the participants’ narratives (Phase 4). During this process, some overlapping themes were merged, while others were divided to better reflect the nuances of the material. The essence of each theme was subsequently refined, and descriptive names were developed to capture the core meaning of the identified patterns (Phase 5). Each theme was defined by its relationship to the overall research question regarding aging in place and social life. This reflexive process ensured that the analysis went beyond mere description and moved toward an interpretative understanding of the participants’ experiences (29). Finally, the analysis was formalized by selecting vivid, representative extracts for the results section. These quotes were integrated with the analytical narrative to demonstrate how the themes were grounded in the data (Phase 6). While the phases of the reflexive thematic analysis are presented sequentially for analytic clarity, the actual process was recursive and overlapping, involving continual engagement with the data, codes, and developing themes.

In line with the reflexive approach to thematic analysis, the coding process moved beyond the manifest content of the interviews to explore latent meanings (29). Rather than merely describing the participants’ explicit statements, the analysis sought to interpret the implicit patterns and underlying assumptions regarding their social lives. This interpretative depth was essential for capturing the nuances of how aging in place with health challenges influences one’s social life and well-being.

Reflexivity was not approached as a strategy to eliminate bias but as an analytic resource that enabled a more critical and nuanced engagement with the data and the meanings constructed through the research process. Throughout the analysis, a reflexive journal was maintained to critically examine preconceptions regarding aging in place and the potential for loneliness among home care recipients. Ongoing critical reflection was used to ensure that the themes derived from the data were not merely reflective of prior expectations but were deeply rooted in the participants’ narrated experiences. This reflexive stance allowed for a more nuanced interpretation of the subtle ways in which social engagement and health challenges intersect for older adults.

The first author held primary responsibility for conducting the analysis; however, close collaboration among all authors was integral to the analytic process and contributed to a broader analytic perspective.

### Ethical considerations

The Regional Committees for Medical and Health Research Ethics in Norway considered that the study fell outside their remit and did not require approval (reference number 398854). The Norwegian Agency for Shared Services in Education and Research approved the study (reference number 689856).

The participants were informed about the study aims and procedures and were assured of confidentiality and anonymity. Written informed consent was obtained prior to participation. At the outset of each interview, participants were reminded of their right to withdraw from the study at any time without any consequences for the care and support they received from home care services. To ensure the participants’ privacy, all potentially identifiable information, including names, places, and dates, was anonymized, and pseudonyms are used.

## Results

A multifaceted picture, where experiences ranged from feelings of belonging and connection to perceptions of loneliness and exclusion, was highlighted. Participants’ accounts were shaped by personal histories, neighborhood dynamics, and broader societal changes.

Three themes were developed to capture the patterns of meaning: (1) Side by side, worlds apart: the pursuit of belonging in changing times; (2) Quality over quantity: prioritizing meaningful connections over social quantity; (3) From ease to effort: navigating barriers to social support. The themes illustrate how social participation is organized in and around the home, influenced by personal dimensions such as health and identity, the relational dynamics of families and friendships, and the broader structural conditions of neighborhoods and communities. They illustrate how participants navigated social life, barriers, and sought meaningful connections in changing times. Each theme is presented in detail below, supported by illustrative excerpts from the interviews. Together, they offer insight into the ways social participation is sustained, or challenged, through ordinary practices and social ties, highlighting its significance for health and well-being in later life.

### Side by side, worlds apart: the pursuit of belonging in changing times

The neighborhood proved to be a central arena for participants’ daily lives and social engagement. Everyday practices such as greeting neighbors or visiting familiar places provided continuity, stability, and a sense of belonging that reinforced identity and security amid aging and broader societal change. For several participants, being part of the same local environment for decades cultivated a deep attachment to place.

For example, Kari, who had lived in the same area for more than 50 years, described her surroundings as an extension of her home. Long-standing relationships with neighbors offered support and trust; she often contributed by baking something to share when receiving visitors and could rely on reciprocal help whenever needed. Olav conveyed a similar sense of inclusion and belonging; caring neighbors maintained social engagement and helped mitigate social isolation and loneliness. Together, these accounts illustrate how the local neighborhood functioned as both a social and emotional anchor, reinforcing a continued sense of self and belonging in later life.

*“All the neighbors here are very nice, and they are all helpful. So that’s good. The man who lives just below here has two children, a boy and a girl. They are so helpful and offer me assistance.” (Olav)*

However, others had the opposite experience regarding their neighborhood. For them, there was a tension between belonging in their neighborhood and not belonging. They expressed a feeling of loss and exclusion. This was particularly evident for some participants, for whom the neighborhood had gradually transformed into a place of social disconnection. For these, the once-familiar environment no longer offered the same sense of belonging, as social networks diminished and everyday encounters became fewer. Gerd’s story exemplifies this shift. Having lived in the same neighborhood for more than 60 years, she was now the only remaining resident from the original community. The surrounding houses were increasingly inhabited by younger families with whom she had limited interaction, reinforcing her sense of social isolation and estrangement. What once represented continuity and shared history had, for her, become a reminder of loss and change, and a feeling of exclusion.

*“Before, we used to talk to each other. I would say the world has completely changed. It’s not like in the old days. But of course, it’s something one must get used to. It’s the same for everyone. That’s how the world is now. They say it’s moving forward, but I’m not so sure it’s for the better.” (Gerd)*

Participants reflected on the transformation of neighborhood dynamics over time. Where once they had known and interacted regularly with their neighbors, many now felt isolated, describing their neighbors as strangers. This shift was experienced as a loss, not only of social contact but also of a more inclusive and supportive environment. The absence of neighborly interaction diminished opportunities for informal support, companionship, and belonging. This experience was echoed by participants living in both rural and urban areas. Even in traditionally close-knit rural communities, participants reported limited contact with neighbors and a lack of inclusion in the local social life.

One participant, Knut, who had relocated from a rural home to an apartment in the municipal center, reported that his sense of loneliness remained unchanged despite the geographical move. Although he was now in closer proximity to neighbors and services, surrounded by others in similar life circumstances, his experience of limited social connection persisted. As he described it:

*“When I live here, I’m alone here too. People do not come to see me often. Occasionally, but nothing more.”* (Knut)

Even though he lived in a more densely populated area, he continued to spend his evenings alone, much as he had in the countryside. Consequently, he had decided to return to his original home. He prioritized a deep sense of belonging and place attachment over the convenience of the center, choosing to return to a rural environment despite its physical isolation from others.

Astrid had relocated to a new area and reported minimal interaction with her neighbors, describing a lack of connection and familiarity.

*“Contact with the neighbors? Never. Maybe once a year.” (Astrid)*

Loneliness was a salient subject among participants, and several acknowledged experiencing moments of feeling lonely. Some reflected on the absence of visits from relatives they had expected, noting that this lack of contact had tangible implications for their well-being. For them, loneliness emerged through a reduction in social connections that might otherwise have enriched their everyday lives and reinforced their sense of belonging.

*“I have enough clothes and I have enough food. Just too few visits.” (Kari)*

Participants cohabiting with a partner described the relationship as a source of significant emotional well-being and support. This companionship provided a foundation for mutual assistance, enabling couples to navigate health-related and daily challenges collectively. Conversely, participants who had lost their spouse particularly missed the companionship and shared routines that had structured their daily lives, elements central to feeling connected to others and to maintaining a sense of belonging in familiar surroundings.

However, it is important to note that not all participants described loneliness as a prominent or pervasive experience. For some, loneliness was portrayed as an intermittent and natural part of life, not something that dominated or defined their overall experience. Although they occasionally felt lonely, they emphasized that it did not fundamentally undermine their broader sense of belonging within their social world or local community.

*“I’m actually lucky. I can just call someone, or something like that, so I’m not as lonely compared to many others that I, or not many, but others that I can compare myself with. I don’t feel very lonely, but sometimes it’s like you feel it, that sometimes I feel lonely and think that, well, well, nobody cares about me. Those are thoughts that come, even though it’s just nonsense.” (Berit)*

The findings suggested that childless participants reported fewer social contacts, making them particularly vulnerable to isolation in advanced age. Lacking intergenerational informal support from children or grandchildren, these participants faced heightened risk as their peer networks naturally diminished over time. Notably, none of the childless participants addressed this absence explicitly; rather, the significance of intergenerational ties emerged through contrast as participants with children frequently highlighted how important their family was for maintaining social engagement and support. As Anne expressed:

*So they visit me a lot, you know, especially my daughter who lives nearby. And my granddaughter comes here every Thursday when she’s in town.*

While these accounts underscore the protective value of family, the impact of childlessness on social connectedness varied across participants’ unique circumstances and broader contextual factors.

### Quality over quantity: prioritizing meaningful connections over social quantity

A central finding in this study was the strong desire for meaningful social participation, particularly that rooted in pre-existing connections and familiarity. The participants emphasized how they valued depth and emotional significance in their relationships rather than the number of social contacts. Meaningful connections were cultivated through interactions that affirmed identity and mutual care, suggesting that the quality of connection is a central resource for well-being in later life.

Regular visits from family members were described as a cornerstone of daily life. They were a meaningful source of joy, safety, and well-being. Ragnhild’s reflections on the meaning of this are echoed in the following:

*“They are also very good at visiting us. Yes, I cannot complain about that. It is so nice to stay in touch, too. I am afraid of losing contact. I am so happy when they come to visit. The one who is here now has two children. One of them has a little one who is almost four, when he comes… they are just so sweet. They say ‘great-grandmother’ and ‘great-grandfather’. Imagine living to be this old.” (Ragnhild)*

Participants emphasized the value they placed on these interactions, which contributed positively to their overall well-being and perceived health. Spending time with family was perceived as meaningful, a source of comfort, conversation, and mutual care. Simply talking together, listening, and feeling included gave participants a sense of belonging and renewed energy in everyday life. The participants not only received practical assistance but also found companionship and a space to discuss the various aspects of life.

Municipally organized social gatherings were appreciated by many participants as they provided opportunities for interaction, conversation, and, in some cases, a shared meal.

*“I think the advantage of living here and having access to what I get every week, namely the day center, is that it’s also very good for my closest relatives that something is happening for Mom now, every Thursday. And it works wonderfully. We get two meals, delicious food, and we sit at a set table, where we have help to sit down at the table. Yes. So, my well-being, that has to do with things like that.” (Inger)*

Some of the participants enjoyed meeting friends, neighbors, and acquaintances through these organized meetings. Particularly events centered around a shared activity were positively received, as they offered a natural context for interaction. However, some participants found such gatherings uncomfortable when they did not know anyone present. The expectation to initiate conversation with strangers could feel awkward and uninviting. One participant, for example, had discontinued her weekly visits to the senior center after the passing of the friends she used to meet there. She no longer felt inclined to engage with unfamiliar people, emphasizing that having someone to talk to, particularly family and close friends, remained a vital and essential aspect of life in older age. Forming new relationships was perceived by some as burdensome and emotionally taxing. In particular, the effort required to establish rapport with new acquaintances was found to be both challenging and draining.

*Not because you want to exclude new people, that’s not it. It just takes a bit more.*

*It takes a bit more. To be kind and friendly, and to listen. Until you get a sense of what kind of person this is. Yes, that’s it. (Liv)*

However, the participants were selective, and they wished to engage socially, but on their own terms. Several participants emphasized the importance of maintaining lifelong friendships. These relationships were valued not only for their longevity but also for the shared history, mutual understanding, familial connections, and continuity they embodied.

*“Yes, it’s something about meeting those you’ve always had. At least the old friends, they are the most important. Because you share common stories with them. Yes, and shared experiences with them.” (Liv)*

Meeting with old friends allowed for immediate connection and was valued as meaningful for the participants, and they expressed a preference for spending time with long-standing friends, former colleagues, and family members, individuals with whom they shared a personal history and familiarity.

### From ease to effort: navigating barriers to social support

Participants described a shift from effortless social engagement earlier in life to increasing challenges in maintaining social connections. Health limitations, mobility constraints, and shrinking social networks required deliberate strategies and persistence, illustrating how preserving social connections becomes an active process under changing circumstances.

Maintaining social connections and interacting with others was described as essential for overall well-being. However, many participants faced substantial barriers to leaving their homes, which limited their opportunities for external social engagement and turned their home into a central place where they lived and maintained their social lives.

The home had become a central arena for social life for several of the participants, and they relied on visits from family, friends, and neighbors. However, for some of the participants, the daily social encounter consisted solely of visits from the home care services. This was particularly evident among those who did not have children, or whose children lived at a distance. With fewer stable opportunities for social interaction, the daily home care visit became an important source of social contact.

Furthermore, participants described a shift in their social engagement, marked by a reduced capacity for participation alongside a continued desire for connection. While many expressed a longing to maintain social ties, the realities of aging and declining health had significantly altered the ways in which they could engage socially. Physical decline, including reduced mobility, incontinence, impaired memory, impaired vision, and hearing loss, was frequently cited as a limiting factor. For instance, reduced vision made it difficult to perceive curbs, which increased participants’ fear and risk of falling and sustaining injuries, while diminished hearing impaired their ability to engage in conversations involving multiple people. Health concerns contributed to social withdrawal in many ways.

*“Well, now because of my leg, which has gotten worse, I’m quite reduced because of that. So, I don’t walk much, no. My balance is poor, and sometimes I take a tumble. I have a mark on my forehead somewhere. It should be a small mark—I fell flat out on the terrace here. Even a small unevenness is enough for me to fall.” (Olav)*

Astrid, for example, postponed social visits while awaiting dental surgery, expressing discomfort about being seen before the procedure.

*“And then it works when the teeth come back in my mouth. There are things I don’t want to join when I don’t have teeth, I can’t take part in those things when they sit and eat. I can’t do that. And I can’t join the exercise sessions, and that’s a sorrow for me, because the balance training was good. I hope they finish the dental work soon; I’ve been waiting for many months.” (Astrid)*

Individuality and heterogeneity in old age were prominent: Participants’ needs and capacities for social engagement varied widely. While some had come to terms with a reduced social life and viewed it as a natural part of aging, others mourned the loss of meaningful social interaction and struggled to accept the change.

*“Social participation. I miss that very much. It’s a big loss.” (Sondre)*

Torill, among others, perceived the reduction in social activity as a natural and manageable transition. Having less energy and prioritizing health and recovery made reduced social participation feel appropriate:

*“Yes, I have probably accepted it. And then it has something to do with age, right? Because when I think about how old I’ve become, is it really meant that one should keep going at the same pace? It’s not that. So, you’re allowed to sit down and maybe fall asleep in the armchair, or read a good book, or just do things you feel like doing that you haven’t had time for before. It’s awful that you have to have a stroke before you do that. Hehe, oh dear.” (Torill)*

Dependence on support further shaped the nature of social participation. The need for help with mobility, transportation, or communication technologies often determined whether and how participants could maintain social contact. For some, adapting to a reduced social life was a pragmatic response to changing circumstances, but for others, it represented a painful disruption to a previously fulfilling aspect of life. Several participants reported a history of active engagement within civil society and various organizations, including consistent involvement in meetings and organizational activities. This activity had ceased due to a combination of factors related to the aging process.

Two participants retained valid driver’s licenses and continued to drive, expressing that they could not envision life without the autonomy driving afforded. For those who had ceased driving, this transition marked a definitive “before and after” in their social lives. The impact was particularly profound for participants residing in rural areas, who described a sudden and significant contraction of their “radius of movement” from one day to the next.

*“The distances become too great to… to the outside world when you no longer have a car. It’s actually a very big turning point in life before and after the driver’s license… I didn’t drive that often, but just having the possibility… To be deprived of a possibility… That is incredibly important for people, I think. It’s about freedom.” (Sondre)*

The shift from ease to effort was also evident in how participants managed family interactions. While those with ample space maintained social ties seamlessly, transitioning to smaller apartments created significant emotional challenges. This loss of “hosting capacity” was not merely a logistical hurdle but a poignant experience that transformed effortless social integration into a strained navigation of support. Similarly, participants lacking familial networks found social participation increasingly effortful as the absence of primary support structures necessitated greater individual exertion to remain connected.

## Discussion

The findings highlighted that how older adults perceived and navigated their social lives was multifaceted, shaped significantly by individual preferences and environmental contexts. While aging often necessitates substantial adjustments to social networks, the participants in this study demonstrated significant resilience. Rather than focusing solely on the limitations of their health or circumstances, they were solution-oriented and maintained a positive outlook regarding their social engagement. While the importance of social participation was universally acknowledged, participants’ preferences for engagement varied considerably. The participants demonstrated a clear desire for meaningful connection, and their experiences and reflections also showed a sense of loss—of community, and of belonging. These findings underscore the importance of recognizing the diversity of older adults’ social needs and the contextual factors that shape their experiences.

Participants exhibited strong emotional engagement with their neighborhoods, although their experiences varied significantly. While some felt a sense of belonging and continuity, others expressed alienation due to generational shifts and lack of contact. What was once a source of comfort and familiarity had become unfamiliar or even isolating, giving the neighborhood a more ambivalent or negative connotation. Notably, this preoccupation was most evident among those who no longer felt at home, illustrating how the local community can serve as both a source of social connection and a space of profound change. This is in line with previous studies that found that neighborhood social cohesion was a strong predictor of well-being among older adults (30, 31). They report that higher social cohesion and a sense of belonging were associated with better physical and social well-being (30). This resonates with the findings in our study. Feeling connected to one’s neighborhood and experiencing a sense of belonging emerged as essential for the participants, consistent with other studies on community-dwelling older adults (24). Perceiving the local environment as unfamiliar or unwelcoming can intensify loneliness and negatively shape well-being for older adults (32, 33). Emerging research in gerontology positions neighborhood social cohesion as an important “upstream” factor for mental health and well-being in later life (31). Rather than being determined solely by individual choices or biological factors, our lives appear to be structurally embedded in the social and environmental context of everyday life. Cohesive neighborhoods, those characterized by trust, mutual support, and shared norms, create a psychosocial climate that shapes how older adults experience aging (31).

Participants emphasized the importance of friends and close, meaningful relationships in their lives. What seemed to matter most was not the number of contacts but the depth and quality of a few trusted relationships. While they actively sought out meaningful interactions, they intentionally chose to forgo gatherings perceived as uninteresting or lacking personal significance. This pattern reflects a broader tendency to prioritize meaningful social connections, suggesting that participation is driven not merely by availability but by the perceived value of the interaction. This resonates with Axén, Christiansen (12) who suggest that a small network of close friends may be more beneficial for well-being than a larger, more superficial social circle. Emotional aspects of relationships are more important than how often older adults have contact with others (34). However, the evidence is not entirely consistent. In a review, Dahlberg, McKee (33) did not find a clear advantage of smaller, intimate networks over larger ones.

The considerable heterogeneity in how loneliness was perceived, combined with the tendency among some participants to avoid explicit discussion of the topic, should not be interpreted as an absence of the phenomenon. Although participants did not always explicitly describe themselves as lonely, some accounts included descriptions of limited social interaction. In several cases, these descriptions appeared alongside explicit statements that participants were not lonely, suggesting that experiences relevant to loneliness may be present without being named as such by participants. Indirect indications of limited social interactions were often expressed through comments about wishing for more visitors, wanting to be able to visit others themselves, or valuing the opportunity to have someone to talk to. This apparent tension between rejecting the label of loneliness while also describing limited social activity informed our interpretation of loneliness as an implicit dimension of some participants’ accounts. Previous research has shown that loneliness can be difficult for older adults to articulate (13, 35–38). It can be challenging to detect because it is a deeply private experience, difficult to verbalize, and closely connected to aging and increasing frailty (13, 35). Moeyersons, De Vliegher (13) described loneliness as an elusive, intangible, and often hidden experience, intertwined with the aging process and shaped by the multiple losses associated with growing older. This can make it difficult to express and may be one explanation.

In the present study, widowed participants were among those who most clearly described feelings of loneliness. This is consistent with previous research showing that bereavement, and particularly the loss of a spouse, substantially increases the risk of loneliness in later life (33, 36). These findings underscore the need for a nuanced assessment that distinguishes chosen solitude from experienced loneliness and for targeted support to those whose social resources have been significantly reduced through loss.

Another noteworthy finding from our study was the extent to which participants expressed satisfaction with their own company and appreciation for solitude or self-chosen loneliness. While this is not a new observation, and has also been highlighted in previous research on aging and well-being (12, 37), it nonetheless offers an important counterpoint to deficit-oriented discourses that equate solitude with loneliness. Several participants described solitude as a valued aspect of daily life, associated with autonomy, reflection, and peace. This is also in accordance with Axén, Christiansen (12) who describe it as a silver lining of being in one’s’ own company and enjoying it. For some, spending time alone was even preferred to socializing, offering opportunities for rest and self-care rather than isolation. These values associated with solitude are also reflected in Tornstam’s theory of gerotranscendence, which emphasizes the positive aspects of being alone as appreciated by older adults (39, 40). Tornstam argues that withdrawal from social or physical activity in later life should not be viewed as disengagement or apathy, but rather as a natural and meaningful form of inner activity. These theoretical insights correspond with the present findings, suggesting that solitude can represent an active and intentional process of self-reflection and personal growth in older age. Such nuances are vital to consider in future service and policy development, as they complicate the current consensus, which focuses predominantly on the negative aspects of solitude, by highlighting the necessity of acknowledging positive dimensions as well.

However, participants underscored the importance of balance. Enjoying solitude did not imply a rejection of social relations; rather, it coexisted with a continued desire for meaningful contact with family and friends. This reflects a nuanced understanding of social well-being in old age, one that acknowledges the coexistence of autonomy and belonging. Recognizing this duality is essential: Solitude can be both a resource and a risk, depending on the person’s context, health, and subjective experience. Supporting older adults thus involves facilitating both opportunities for social participation and respect for the individual’s need for privacy and autonomy. There is a risk that valued solitude can tip over into unwanted loneliness, particularly when opportunities for meaningful interaction are reduced. This is especially salient for older adults living alone, who are consistently identified as being at heightened risk of loneliness (41). One study also suggested that older adults may overstate the positive aspects of being alone as a coping strategy, both to avoid worrying their close relatives and to avoid being perceived as a burden (13, 36).

Among participants in this study, some demonstrated considerable agency, taking initiative to organize regular activities and social contact, while others exhibited more passive patterns of engagement. These variations must be understood not merely as personal dispositions but as situated within differing social, material, and emotional resources. For some of the oldest participants, the death of peers and a shrinking social network left limited opportunities for continued participation. Others appeared to have less social capital, making it difficult to sustain relationships or access social arenas. It is evident that individuals hold some degree of personal responsibility for maintaining a social life. However, this responsibility cannot be understood in isolation from one’s life history and social context. For those who have not been socially active earlier in life, aging may amplify patterns of limited participation, particularly as social arenas become fewer. Retirement, adult children moving out, and the absence of workplace-based interactions can all contribute to reduced opportunities for informal social contact. Our study found that family and friends were particularly important for the participants. This corresponds with Townsend, Chen (24), who reported that maintaining social connections in old age is often facilitated by pre-existing networks of family and friends. The ability to “take responsibility” for one’s social life is mediated not only by motivation or willingness but also by bodily capacities, access to enabling environments, and cultural norms around aging, independence, and dignity. A nursing and health promotion perspective must therefore attend to how social participation is conditioned by both internalized constraints and external structural factors.

### Limitations

A limitation of this study relates to data repurposing. Because the original interview guide did not explicitly target social dynamics or loneliness, the degree of follow-up probing on these specific themes varied across the 24 accounts. This inconsistency may have restricted the narrative depth, detail, and comparability of participants’ experiences. Consequently, these contextual constraints should be considered when interpreting the themes, particularly regarding the nuances of social connections.

## Conclusion

Supporting social engagement in later life necessitates more than merely encouraging individual initiative; it requires a profound recognition of the complex, lived realities that shape agency. These findings suggest that while current aging policies advocate for autonomy and self-determination, they often overlook the personal, relational, and contextual constraints that define the aging experience. Consequently, a more nuanced understanding of aging in place must account for the fundamental interdependence between individual capacity and the social conditions that sustain it. Ultimately, enhancing the well-being of older adults depends on the structural facilitation of active social lives, ensuring that environments are designed to actively cultivate rather than merely permit connection.

To refine future practice, further research is essential to distinguish between desired solitude and undesired loneliness from the perspective of older adults themselves. This study underscores that personal experiences often diverge from prevailing political and societal discourses. Ultimately, bridging this gap is crucial for developing policies that are not only theoretically sound but also deeply resonant with the heterogeneous needs and perspectives of an aging population.

## Data Availability

The data are not publicly available due to individuals’ privacy under the European General Data Protection Regulation and ethical restrictions. Requests to access the datasets should be directed to Therese.hugoy@ldh.no.
